# Reproducibility of ^18^F-fluoromisonidazole intratumour distribution in non-small cell lung cancer

**DOI:** 10.1186/s13550-016-0210-y

**Published:** 2016-11-07

**Authors:** Milan Grkovski, Jazmin Schwartz, Andreas Rimner, Heiko Schöder, Sean D. Carlin, Pat B. Zanzonico, John L. Humm, Sadek A. Nehmeh

**Affiliations:** 1 Department of Medical Physics, Memorial Sloan Kettering Cancer Center, 1275 York Avenue, New York, NY 10065 USA; 2 Department of Radiation Oncology, Memorial Sloan Kettering Cancer Center, New York, NY USA; 3 Department of Radiology, Memorial Sloan Kettering Cancer Center, New York, NY USA; 4 National Center for Cancer Care and Research, Doha, Qatar

**Keywords:** Non-small cell lung cancer, Hypoxia, ^18^F-fluoromisonidazole, Reproducibility, Quantification

## Abstract

**Background:**

Hypoxic tumours exhibit increased resistance to radiation, chemical, and immune therapies. ^18^F-fluoromisonidazole (FMISO) positron emission tomography (PET) is a non-invasive, quantitative imaging technique used to evaluate the presence and spatial distribution of tumour hypoxia. To facilitate the use of FMISO PET for identification of individuals likely to benefit from hypoxia-targeted treatments, we investigated the reproducibility of FMISO PET spatiotemporal intratumour distribution in patients with non-small cell lung cancer (NSCLC).

**Methods:**

Ten patients underwent ^18^F-fluorodeoxyglucose (FDG) PET/CT scans, followed by two FMISO PET/CT scans 1–2 days apart. Nineteen lesions in total were segmented from co-registered FDG PET image sets. Volumes of interest were also defined on normal contralateral lung and subscapularis muscle. The Pearson correlation coefficient *r* was calculated for mean standardized uptake values (SUV) within investigated volumes of interest and for voxels within tumour volumes (*r*_TV_). The reproducibility of FMISO voxelwise distribution, SUV- and tumour-to-blood ratio (TBR)-derived indices was assessed using correlation and Bland-Altman analyses.

**Results:**

The SUV_max_, SUV_mean_, TBR_max_, and TBR_mean_ were highly correlated (*r* ≥ 0.87, *p* < 0.001) and were reproducible to within 10–15 %. The mean *r*_TV_ was 0.84 ± 0.10. 77 % of voxels identified as hypoxic on one FMISO scan were confirmed as such on the other FMISO scan. Mean voxelwise differences between TBR values as calculated from pooled data including all lesions were 0.9 ± 10.8 %.

**Conclusions:**

High reproducibility of FMISO intratumour distribution in NSCLC patients was observed, facilitating its use in determining the topology of the hypoxic tumour sub-volumes for dose escalation, in patient stratification strategies for hypoxia-targeted therapies, and in monitoring response to therapeutic interventions.

**Trial registration:**

Current Controlled Trials NCT02016872

## Background

Non-small cell lung cancer (NSCLC) remains the leading cause of cancer-related mortality worldwide [1]. Tumour-cell hypoxia, a common feature of solid tumours, is a pivotal determinant of the effectiveness of radiation, chemical, and immune therapies and is associated with poor overall survival [2, 3]. The hypoxic microenvironment can be assessed by a variety of approaches, e.g. by measurement of partial pressure of oxygen with polarographic electrodes [4] or by immunohistochemical detection of endogenous and exogenous hypoxia markers [5]. However, such procedures are invasive and potentially hazardous, restricted to accessible lesions, and limited by sampling errors.

^18^F-fluoromisonidazole (FMISO) positron emission tomography (PET), a non-invasive imaging technique, presents an attractive alternative [6–8]. FMISO is clinically the most extensively investigated hypoxia PET tracer. Several studies in lung cancer patients have suggested stratification strategies based on FMISO uptake and kinetics [9–12]. The hypothesis is that selective dose painting of putative radioresistant hypoxic tumour sub-volumes, as defined by FMISO PET, may improve locoregional control [13]. Numerous efforts also continue to evaluate ^18^F-fluorodeoxyglucose (FDG) PET for target delineation in radiotherapy [14, 15] and explore the utility of intensity-modulated radiotherapy (IMRT) based on FDG voxel intensities [16, 17]. However, despite a number of ongoing hypoxia-imaging trials, quantitative assessment of intratumour distribution of hypoxia-specific PET tracers has yet to be widely implemented in the clinical decision-making process.

In order to fully exploit the benefits of incorporating tumour hypoxia information as obtained by FMISO PET into patient management, whether as an IMRT target, in patient stratification strategies for hypoxia-targeting regimens, or for monitoring response to therapeutic interventions, it is essential to examine the spatiotemporal reproducibility of FMISO intratumour distribution. To our knowledge, such studies have been performed only in head and neck cancer (HNC) patients, with discordant results [18, 19]. Due to the absence of similar studies in other tumour entities, e.g. lung cancer, it remains unclear to what extent the reproducibility of FMISO will be affected by the lack of rigid immobilization and the presence of respiratory motion. Therefore, the aim of this pilot study was to investigate the reproducibility of FMISO intratumoural distribution in serial baseline FMISO PET scans in a cohort of NSCLC patients.

## Methods

### Ethics statement

This study was approved by Memorial Sloan Kettering Cancer Center’s Institutional Review Board (IRB 13-186; registered under www.clinicaltrials.gov identifier number NCT02016872), and all subjects signed a written informed consent regarding the examination and use of anonymized data for research and publication purposes. The methods were carried out in accordance with the approved guidelines.

### Patient characteristics

The eligibility criteria were as follows: age > 18 years, pathological confirmation of NSCLC, no prior treatment, primary or nodal tumour measuring ≥2 cm on CT, and a Karnofsky performance status of ≥70 %. Exclusion criteria included pregnant or breast-feeding women and patients with severe diabetes (fasting blood glucose >200 mg/dl). Fifteen patients agreed to participate in the study. Patients were scanned on a flat-top couch insert and immobilized in an alpha cradle (Smithers Medical Products, Inc.). As the second FMISO PET scan was not acquired for five patients due to their inability to continue (*n* = 3) or technical reasons (*n* = 2), ten patients in total were included in the reproducibility analysis (Table 1).

**Table 1:** Patient characteristics

Patient	Age (years)	Gender	Stage^a^	Histopathology
1	63	F	IV	Adenocarcinoma
2	75	F	IIA	Adenocarcinoma
3	72	F	IB	Squamous cell carcinoma
4	31	M	IIIB	Adenocarcinoma
5	63	M	IIIB	Adenocarcinoma
6	77	M	IIIB	Squamous cell carcinoma
7	70	M	IIA	Squamous cell carcinoma
8	56	F	IIIB	Adenocarcinoma
9	59	M	IIIA	Adenocarcinoma
10	73	M	IIIA	Adenocarcinoma

^a^American Joint Committee on Cancer 7th Edition

### ^18^F-fluorodeoxyglucose PET/CT protocol

Each patient underwent an FDG PET/CT study scan for radiotherapy simulation purposes. Patients were injected intravenously with 460 ± 17 MBq (range, 429–477 MBq) of FDG, after a fasting period of ≥6 h. PET scans were acquired for 3 min/bed position, at 100 ± 38 min (range, 60–171 min) post-injection (pi). All PET data were acquired in 3D mode on a General Electric Discovery ST PET/CT (GE Health Care Inc.). A CT acquired in cine mode (140 kVp, 10 mA, 5.0-mm slice thickness, 0.5-s tube rotation) was averaged (CT_avg_) and used for attenuation correction of PET images. The cine duration was set to match the patient breathing period plus 1 s (~6 s on average). PET emission data were corrected for attenuation, scatter, and random events and reconstructed into 128 × 128 × 47 matrix (voxel dimensions 5.47 × 5.47 × 3.27 mm). The reconstruction was performed using the GE ordered subset expectation maximization (OSEM) algorithm with standard clinical reconstruction parameters: 2 iterations, 16 subsets, and 6.0 mm full width at half maximum Gaussian post-filter.

### ^18^F-fluoromisonidazole PET/CT protocol

Ten patients underwent two FMISO PET studies each (i.e. FMISO1 and FMISO2). FMISO1 was performed 2.4 ± 1.4 days (range, 1–5 days) after the FDG PET/CT, with FMISO2 being performed 1.7 ± 1.6 days (range, 1–6 days) after FMISO1. Patients received an average FMISO bolus injection of 388 ± 15 MBq (range, 356–407 MBq). Data were acquired for 10 min over one field of view (~15 cm; centred on the lesions) at 163 ± 13 min pi (range, 144–183 min). A low-dose cine CT scan (the same parameters as for the FDG study) was performed and used for attenuation correction and image co-registration. All FMISO PET images were reconstructed using OSEM with the standard clinical parameters.

### Image analysis

The FDG and FMISO2 tumour volumes were co-registered to those of FMISO1 by means of their corresponding CT_avg_ image sets, using the GE AW Workstation v4.6 General Co-Registration tool (GE Health Care Inc.). Rigid transformation was used, and the results were visually inspected for potential mismatches. The transformation matrices obtained were then applied to the corresponding PET images. Tumour metabolic target volumes (TV) were delineated on the FDG PET images with the adaptive threshold algorithm in the GE AW Workstation PET VCAR™ (Volume Computer-Assisted Reading) semi-automated software (FDA-approved), which is based on the companion CT as a fiduciary marker and a count-based edge recognition algorithm. The corresponding target volumes were subsequently copied onto the two co-registered FMISO image sets.

Tumour uptake in the target volumes on the two FMISO scans was compared on a voxel-by-voxel basis in PMOD v3.604 (PMOD Technologies GmbH). Activity concentration data were converted into standardized uptake values (SUV; normalized to lean body mass). The blood SUV (SUV_blood_) was measured by (i) segmenting the descending aorta on the CT_avg_, (ii) copying the volume of interest (VOI) to the corresponding FMISO PET, (iii) eroding the VOI by 1 voxel in 3D, and (iv) measuring the average SUV within the eroded VOI.

Hypoxic sub-volume (HTV; in cm^3^) was defined as including voxels within the TV having a tumour-to-blood ratio (TBR) ≥ 1.2 on both FMISO scans [8]. For each esion, maximum and mean values for voxels within the TV were calculated in units of SUV (SUV_max_, SUV_mean_) and TBR (TBR_max_, TBR_mean_). Reproducibility of FMISO uptake was also assessed in the non-diseased normal lung tissue (by evaluating the mean SUV within a 20-mm-diameter spherical VOI that was placed in the healthy contralateral lung; SUV_lung_) and in the non-diseased muscle (by evaluating the mean SUV within a manually drawn VOI on the subscapularis muscle on the CT_avg_ and subsequently copied to the corresponding PET FMISO scan; SUV_muscle_).

### Statistical analysis

The Pearson correlation coefficient was calculated between the FMISO1 and FMISO2 intratumour distributions (*r*_TV_) and between all SUV- and TBR-derived indices. The normality of the distribution of differences in the investigated parameters between the two FMISO studies was verified with a two-sample Kolmogorov-Smirnov test. This was done to validate the applicability of Bland-Altman analysis, which was subsequently performed to calculate the mean differences between voxelwise TBR values and 95 % limits of agreement (LoA) [20]. The latter are defined as ±1.96 * SD of the mean differences and represent the boundaries within which 95 % of observations are expected to be observed. *p* < 0.05 was assumed to represent statistical significance. To evaluate the geographic stability of hypoxic sub-volumes, the percentage of intratumour voxels that were identified as hypoxic in both FMISO studies was calculated, as based on the TBR ≥ 1.2, ≥1.4, and ≥1.6 thresholds [6–9]. All statistical analyses were carried out in MedCalc v15.6 (MedCalc Software bvba).

## Results

Nineteen FDG-avid lesions were included in the analysis. None of the investigated lesions were located near the edge of the PET field of view. The average lesion volume was 28 cm^3^ (range, 4–111 cm^3^). No lesions were found that would exhibit uptake on the FMISO scan while being negative on the corresponding FDG scan. As mismatches between PET and CT scans were identified in two patients (#4 and #9), the co-registrations were modified manually based on the PET images. All differences between the FMISO1 and FMISO2 scans in the SUV- and TBR-derived parameters, imaging time pi, and injected dose were normally distributed, as assessed with Kolmogorov-Smirnov test (*p* > 0.05). Tumour volume, imaging time pi, SUV_blood_, SUV_lung_, SUV_max_, SUV_mean_, TBR_max_, TBR_mean_, HTV, and *r*_TV_ are summarized in Table 2. Significantly high correlations were observed between all SUV- and TBR-derived parameters from the first and second FMISO scans (*r* ≥ 0.87, *p* < 0.001) and HTV (*r* = 0.99, *p* < 0.001).

**Table 2:** Summary of FMISO PET reproducibility analysis in patients with non-small cell lung cancer

Patient (lesion)	TV (cm^3^)	Scan time pi (min)		SUV_lung_		SUV_muscle_		SUV_max_		SUV_mean_		TBR_max_		TBR_mean_		HTV (cm^3^)		*r*_TV_
		FMISO1	FMISO2	FMISO1	FMISO2	FMISO1	FMISO2	FMISO1	FMISO2	FMISO1	FMISO2	FMISO1	FMISO2	FMISO1	FMISO2	FMISO1	FMISO2	
1 (1)	9.7	146	144	0.31	0.34	0.86	0.86	1.31	1.13	0.92	0.83	1.53	1.19	1.07	0.87	3.0	0.0	0.83
2 (2)	36.6	153	150	0.25	0.26	0.94	0.94	1.86	1.90	1.18	1.34	1.97	1.92	1.25	1.36	19.6	25.7	0.93
3 (3)	15.6	175	178	0.37	0.31	0.94	1.01	1.34	1.52	1.05	1.06	1.37	1.20	1.06	1.02	1.8	0.1	0.72
4 (4)	41.6	179	183	0.38	0.39	1.28	1.20	1.52	1.62	1.26	1.18	1.50	1.56	1.13	1.14	13.8	13.7	0.52
4 (5)	20.1							1.64	2.11	1.23	1.42	1.50	2.02	1.13	1.27	7.7	11.1	0.83
5 (6)	52.3	171	176	0.16	0.19	1.14	1.22	2.05	2.08	1.35	1.49	1.56	1.48	1.03	1.07	9.2	10.0	0.87
5 (7)	33.5							1.95	2.00	1.28	1.39	1.48	1.44	0.97	1.00	5.8	7.8	0.95
5 (8)	7.0							1.15	1.24	0.92	1.02	0.88	0.89	0.70	0.73	0.0	0.0	0.92
6 (9)	110.6	161	165	0.36	0.37	1.03	1.08	1.50	1.58	1.20	1.27	1.50	1.46	1.20	1.18	65.1	56.9	0.80
7 (10)	19.4	156	158	0.32	0.31	1.04	0.98	1.51	1.35	1.19	1.11	1.24	1.17	0.97	0.96	0.3	0.0	0.87
7 (11)	7.5							1.40	1.52	1.15	1.21	1.15	1.31	0.94	1.04	0.0	1.0	0.91
8 (12)	31.6	166	182	0.40	0.38	0.91	0.87	1.42	1.34	0.98	0.99	1.41	1.38	0.97	1.02	1.5	2.3	0.72
8 (13)	61.0							2.19	1.85	1.25	1.21	2.16	1.91	1.23	1.25	30.7	32.6	0.92
8 (14)	17.1							1.90	1.85	1.15	1.11	1.88	1.91	1.14	1.14	6.7	6.8	0.94
8 (15)	4.1							1.05	0.87	0.74	0.69	1.04	0.90	0.73	0.71	0.0	0.0	0.83
9 (16)	23.5	165	148	0.32	0.27	0.91	0.83	1.22	0.91	0.90	0.64	1.11	0.96	0.81	0.68	0.0	0.0	0.81
10 (17)	12.9	164	147	0.28	0.31	1.01	1.06	1.12	1.20	0.83	0.94	1.29	1.30	0.95	1.02	0.7	1.9	0.90
10 (18)	8.1							0.98	1.12	0.79	0.85	1.13	1.21	0.91	0.92	0.0	0.0	0.88
10 (19)	10.1							0.93	1.06	0.72	0.84	1.06	1.15	0.82	0.91	0.0	0.0	0.80
Maximum	110.6	179	183	0.40	0.38	11.28	1.22	2.19	2.09	1.35	1.49	2.16	2.02	1.25	1.36	65.1	56.9	0.95
Minimum	4.1	146	144	0.16	0.19	0.82	0.83	0.93	0.87	0.72	0.64	0.88	0.89	0.70	0.68	0.0	0.0	0.52
Mean ± SD	27.5 ± 25.7	164 ± 10	163 ± 16	0.32 ± 0.07	0.31 ± 0.06	1.00 ± 0.13	1.01 ± 0.14	1.48 ± 0.37	1.49 ± 0.39	1.06 ± 0.20	1.08 ± 0.25	1.41 ± 0.33	1.39 ± 0.35	1.00 ± 0.16	1.02 ± 0.19	9 ± 16	9 ± 15	0.84 ± 0.10
Pearson correlation		0.77		0.91		0.89		0.89		0.90		0.87		0.90		0.99		

**Fig. 1:**
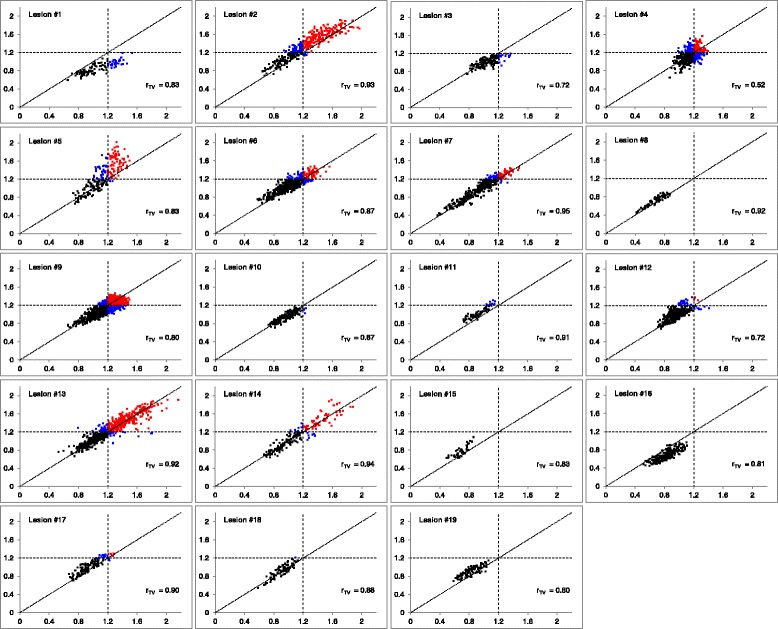
Reproducibility of FMISO intratumor distribution in patients with NSCLC. Voxelwise scatter plots of tumour-to-blood ratio in FMISO1 (*x-axis*) vs. FMISO2 (*y-axis*) are presented for all 19 lesions. *Black*, *blue*, and *red voxels* represent normoxic, hypoxia-ambiguous, and hypoxic tumour sub-volumes, respectively, as based on the TBR ≥ 1.2 threshold (*dashed lines*). Equality lines (*dotted*) and *r*_TV_ are also displayed for all scatter plots. *r*_TV_ values were significant in all cases

Scatter plots for the co-registered FMISO images display intratumour voxels colour-coded according to their hypoxia status as based on the TBR ≥ 1.2 threshold (Fig. 1). The mean *r*_TV_ was 0.84 ± 0.10 (range, 0.52–0.95), with all lesions except for one having *r*_TV_ > 0.70. The hypoxic status (i.e. the presence of intratumour voxels with TBR above the pre-defined threshold) remained unchanged in nine out of ten patients between the two scans, regardless of the implemented threshold. 77 %, 68 %, and 63 % of voxels identified as hypoxic on one FMISO scan were confirmed as such on the other FMISO scan (based on TBR ≥ 1.2, ≥1.4, and ≥1.6, respectively).

**Fig. 2:**
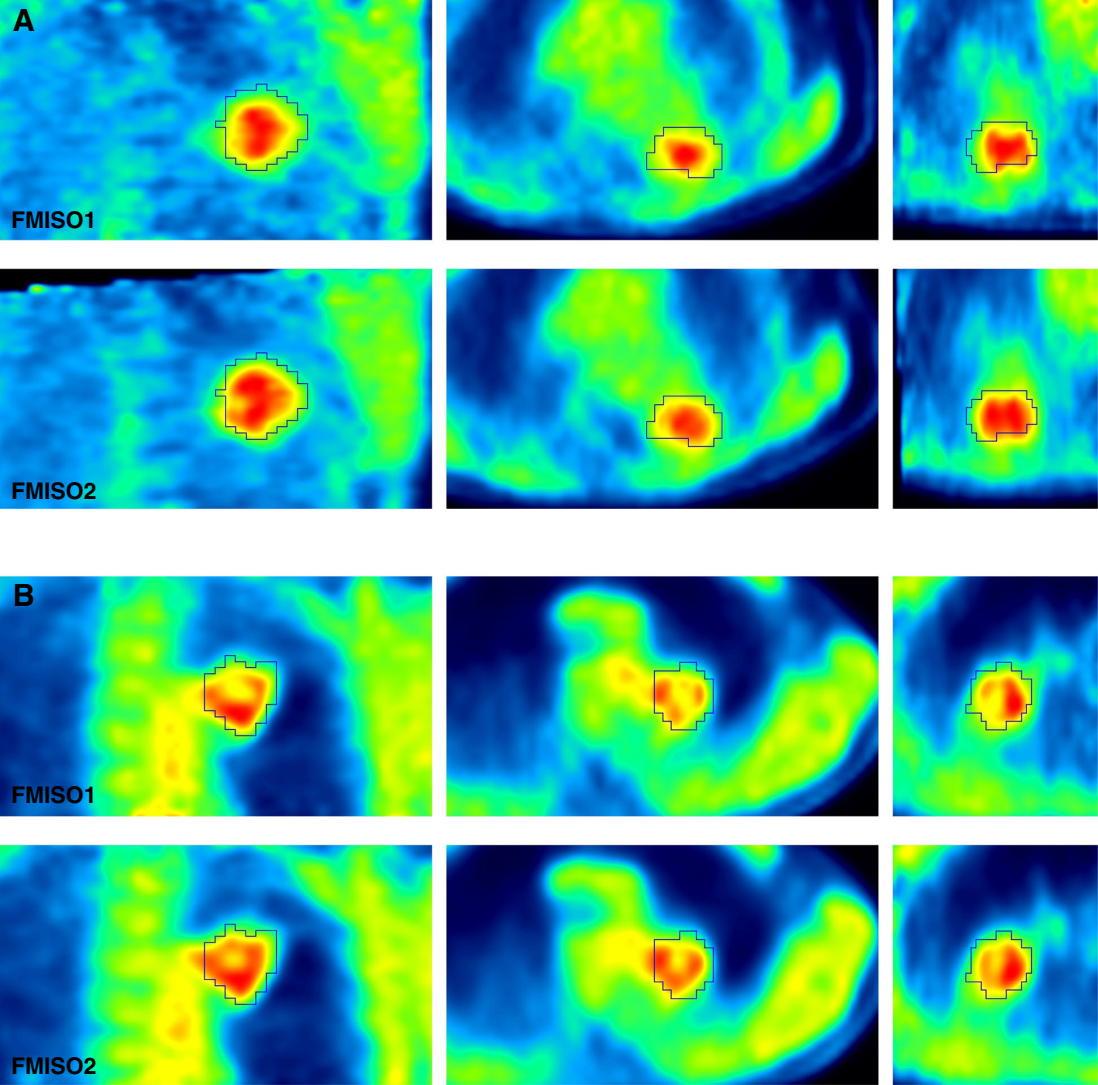
FMISO PET images of two patients with non-small cell lung cancer. From *left* to *right*: coronal, axial, and sagittal slices showing the first (*upper row*) and second (*lower row*) FMISO PET scans of **a** patient #2 (lesion #2) and **b** patient #5 (lesion #7). PET images are windowed at 0–1.8 (**a**) and 0–1.4 (**b**) tumour-to-blood ratio, respectively

No significant correlation could be established between SUV_lung_ or SUV_muscle_ and SUV_max_, SUV_mean_, TBR_max_, or TBR_mean_. The muscle-to-blood ratio, defined as SUV_muscle_/SUV_blood_, was 0.97 ± 0.11 across the patients, confirming that FMISO uptake for non-diseased normoxic tissues approaches unity. Representative FMISO PET/CT images from both scans are displayed for two patients (Fig. 2).

**Fig. 3:**
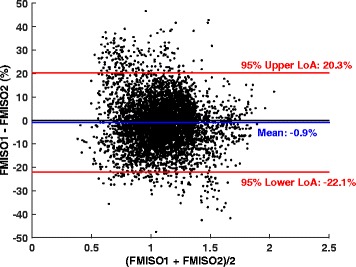
Bland-Altman analysis results for pooled data from all 19 lesions. Relative differences in voxelwise TBR values are shown against the average value combined from the two FMISO scans. Mean and both upper and lower limits of agreement (*LoA*) are displayed as *red* and *blue lines*, respectively

Bland-Altman analysis revealed that voxelwise SUV- and TBR-derived indices were reproducible to within 8–13 %, as calculated from 95 % confidence intervals of the mean differences for each lesion (Table 3). Limits of agreement were between 12 % (SUV_muscle_) and 27 % (SUV_max_). Percentages are reported instead of absolute values, to facilitate direct comparison of results across different indices. Voxelwise Bland-Altman analysis revealed an average relative difference of 0.9 ± 10.8 % between FMISO1 and FMISO2, as calculated from pooled data including all 19 lesions (*n* = 5320 voxels; Table 4, Fig. 3). The associated limits of agreement indicate that for 95 % of cases, the relative differences will be within 22 %.

**Table 3:** Bland-Altman analysis results for all image-derived features

	KS test (*p* value)	Mean ± SD (95 % CI) (%)	LLA, ULA (%)
SUV_blood_	0.97	1 ± 8 (−5 to 7)	−15, 17
SUV_max_	0.96	−1 ± 13 (−6 to 7)	−27, 25
SUV_mean_	0.96	1 ± 11 (−4 to 7)	−21, 24
TBR_max_	1.00	−2 ± 12 (−7 to 4)	−25, 22
TBR_mean_	0.96	1 ± 9 (−3 to 5)	−16, 18
SUV_lung_	0.96	0 ± 10 (−7 to 7)	−19, 20
SUV_muscle_	0.98	0 ± 6 (−4 to 4)	−12, 12

*KS* Kolmogorov-Smirnov, *CI* confidence interval, *LLA* lower limit of agreement, *ULA* upper limit of agreement

**Table 4:** Bland-Altman analysis results for differences between voxelwise tumour-to-blood ratio values

Lesion #	Mean ± SD (95 % CI)	LLA, ULA
1	−0.21 ± 0.12 (−0.45 to 0.04)	−0.45, 0.04
2	0.11 ± 0.10 (0.10 to 0.12)	−0.09, 0.31
3	−0.04 ± 0.09 (−0.05 to −0.03)	−0.21, 0.13
4	−0.01 ± 0.13 (−0.03 to 0.00)	−0.26, 0.23
5	0.14 ± .19 (0.11 to 0.17)	−0.23, 0.51
6	0.04 ± 0.09 (0.03 to 0.05)	−0.13, 0.21
7	0.04 ± 0.07 (0.03 to 0.05)	−0.11, 0.18
8	0.04 ± 0.04 (0.03 to 0.05)	−0.04, 0.13
9	−0.03 ± 0.09 (−0.03 to −0.02)	−0.20, 0.15
10	−0.01 ± 0.06 (−0.02 to −0.01)	−0.12, 0.09
11	0.10 ± 0.05 (0.09 to 0.11)	0.00, 0.20
12	0.05 ± 0.09 (0.04 to 0.06)	−0.13, 0.24
13	0.01 ± 0.11 (0.00 to 0.02)	−0.20, 0.22
14	0.01 ± 0.10 (−0.01 to 0.02)	−0.19, 0.20
15	0.09 ± .07 (0.07 to 0.11)	−0.05, 0.22
16	−0.13 ± 0.07 (−0.14 to −0.12)	−0.28, 0.01
17	0.07 ± 0.07 (0.06 to 0.08)	−0.06, 0.20
18	0.01 ± 0.07 (0.00 to 0.02)	−0.12, 0.14
19	0.08 ± 0.07 (0.07 to 0.10)	−0.05, 0.22
Pooled data	0.01 ± 0.12 (0.01 to 0.02)	−0.22, 0.24

*CI* confidence interval, *LLA* lower limit of agreement, *ULA* upper limit of agreement

## Discussion

It is important to determine the reproducibility of image-based prognostic and predictive parameters, including those deduced from nuclear medicine images, which typically exhibit greater statistical variation (i.e. noise) than other modalities. This is especially true for hypoxia-selective radiotracers such as FMISO in light of its generally low tumour uptakes and tumour-to-background ratios. An evaluation of the reproducibility of FMISO PET in NSCLC is a prerequisite if FMISO images are to be rationally used in stratification of NSCLC patients for hypoxia-targeting regimens, monitoring response to therapeutic interventions, or to determine the topology of the hypoxic tumour sub-volumes for dose escalation.

Our data showed strong correlations for both SUV- and TBR-derived metrics between repeated FMISO scans, corroborating results from FDG and ^18^F-flortanidazole (HX4) PET scans of NSCLC patients [21–23]. TBR_max_ and TBR_mean_ were as reproducible as SUV_max_ and SUV_mean_, despite the fact that the definition of a second region of interest to measure the blood activity introduces additional source of variability. The classification status (i.e. indicating either the presence or absence of tumour hypoxia) as based on one FMISO scan remained unchanged in the majority (9/10) of patients when reassessment was performed using the other FMISO scan. These results are encouraging in the context of using FMISO PET in stratification of NSCLC patients for hypoxia-targeted treatments. Changes in FMISO uptake were reported to measure the early response to chemoradiotherapy in NSCLC [10]; however, it remains unclear to what extent the spatiotemporal variability of FMISO PET affects the quality of monitoring treatment response.

Data on the reproducibility of FMISO intratumour distributions from serial FMISO PET scans have been presented previously only for HNC patients, in two separate studies by Nehmeh et al. [18] followed by Okamoto et al. [19]. While Nehmeh et al. reported variability in spatial uptake, speculating that the possible differences were observed due to transient hypoxia, Okamoto et al. subsequently showed that FMISO intratumour distributions were highly reproducible. These contradictory observations may be attributable to (i) imaging at different times post-injection (162 ± 21 vs. 262 ± 21 min), (ii) differences between scan times post-injection for the two FMISO studies (13 ± 8 vs. 3 ± 3 %), (iii) different acquisition times and modes (8 min in 2D mode vs. 10 min in 3D), (iv) different PET/CT cameras (GE Discovery LS with an axial field of view of 15.2 cm vs. newer generation Siemens TruePoint Biograph with an axial field of view of 21.6 cm), and (v) different co-registration algorithms used in the studies by Nehmeh et al. and Okamoto et al., respectively.

Okamoto and colleagues further speculated that another potential reason for the discrepancy between the two studies might have been imaging time post-injection and considered imaging at 4+ h to be more suitable, due to slow clearance of FMISO from the blood [19]. While longer waiting periods should in principle increase the contrast (and image noise), our results indicate that for non-small lung cancer, similarly high reproducibility can be obtained when imaging already at 2.5 h post-injection. The mean *r*_TV_ (0.84 ± 0.10) is comparable to the results from Okamoto et al. (0.89 ± 0.09 [19]), though not with those from Nehmeh et al. (0.60 ± 0.14 [18]). While in the current study patients were imaged at 163 ± 13 min pi, there are several differences in the methodology compared to that by Nehmeh and colleagues [18]: (i) variations in scan times were substantially lower (5 ± 4 %), (ii) data were acquired in 3D mode for 10 min, (iii) image acquisition was performed on a more recent version of the GE PET/CT scanner, and (iv) a different (FDA-approved) image co-registration software was used compared to the previous study which utilized in-house image co-registration software [18]. The quality of co-registrations may have additionally affected the voxelwise correlation (for example, deliberate misregistration by a single voxel in patient #3 resulted in a drop of *r*_TV_ from 0.72 to 0.17).

More recently, reproducibility of hypoxia imaging using HX4 PET has been investigated by Zegers and colleagues in a multicenter trial in both HNC and NSCLC patients [23]. The authors concluded that HX4 PET imaging is reproducible regarding the spatial uptake in both HNC and NSCLC patients, reporting no major differences in the results between the two cohorts [23]. The mean ΔSUV was 0.02 ± 0.07; high correlations were reported between SUV_max_ and TBR_max_ as well as between hypoxic sub-volumes [23]. Our results are also in concordance with this study.

Scatter plots indicate systematic differences in voxelwise uptake between the two FMISO scans, also observed in earlier PET reproducibility studies [18, 19, 23]. Various technical (e.g. incorrect synchronization of time between injection and calibration), biologic (uptake period, presence of acute hypoxia, patient motion, breathing, and comfort), and physical factors (VOI for the calculation of SUV_blood_) might affect PET quantification [24]. However, the mean difference in voxelwise TBR values from pooled data was 0.9 ± 10.8 %, suggesting no systematic biases. This observation is further supported by the absence of significant correlation between SUV values in normal tissues (contralateral lung and subscapularis muscle) and in the tumour. Approximately 23 % of voxels identified as hypoxic on one FMISO scan did not meet the hypoxia criterion on the other FMISO scan (assuming the TBR > 1.2 threshold). In addition to the aforementioned factors, this could be attributed to relatively low uptake of FMISO that exacerbates the impact of statistical noise, potential mismatch between the PET and the CT images (affecting attenuation correction), CT-CT co-registration of the FMISO1 to FMISO2 image sets, and/or the susceptibility of the lesion to respiratory motion due to its location within the lung. Resampling of FMISO2 resulted on average in <3 % absolute differences in uptake values. When correlation analysis was repeated by co-registering FMISO1 to FMISO2, the change in *r*_TV_ was <1 % (data not shown). The extent to which the changes in spatial distribution of tumour hypoxia compromise the coverage of hypoxic tumour sub-volumes achievable by IMRT remains to be investigated.

A limitation of the current study is a small sample size. Nevertheless, high reproducibility of FMISO spatiotemporal distribution was confirmed, providing an impetus for the use of FMISO PET imaging in thoracic oncology. Another limitation of this as well as earlier PET reproducibility studies in NSCLC is the absence of respiratory gating [21–23]. While motion correction is not yet widely used clinically [22], it may alter the accuracy of quantitative uptake measures due to image blurring [25]. However, similar reproducibility of FMISO was observed for non-small cell lung cancer patients as for patients with head and neck cancer [19], despite the fact that the latter were immobilized during image acquisition, the tumours were not affected by respiratory motion, and for which the co-registration is expected to be more accurate. Lastly, the clinical significance of the observed variability in FMISO intratumour distribution in the context of patient stratification for hypoxia-targeting therapies, monitoring treatment response, efficacy of biologically conformal radiotherapy, or radiomics warrants further examination in larger datasets.

## Conclusions

The results of this pilot study confirm that (i) FMISO intratumour distribution is highly reproducible in NSCLC, facilitating its use in dose escalation of hypoxic tumour sub-volumes, patient stratification strategies, and monitoring treatment response; (ii) high reproducibility can be achieved with relatively shorter imaging times post-injection than those previously suggested, potentially reducing long patient waiting periods; and (iii) the spatiotemporal uptake patterns of FMISO as measured by PET are not expected to be affected by transient hypoxia.
